# SIRPα Immuno-PET Provides a Pan-Myeloid View of Immune Activity During COVID-19

**DOI:** 10.2967/jnumed.125.270781

**Published:** 2026-01

**Authors:** Sridhar Nimmagadda

**Affiliations:** Russell H. Morgan Department of Radiology and Radiological Science, Johns Hopkins University School of Medicine, Baltimore, Maryland

The coronavirus disease 2019 (COVID-19) pandemic underscored the need for the development of tools that can visualize immune system activity in the context of viral inflammation. Although [^18^F]FDG PET can reveal sites of heightened metabolism (1), and translocator protein (TSPO) tracers capture certain activated macrophage states (2), these tools provide only fragments of a much larger picture. What has been missing is a way to map the distribution and persistence of myeloid cells (monocytes, macrophages, dendritic cells, and granulocytes) across the whole body. Addressing this gap matters because dysregulated myelopoiesis, immature monocyte influx, and neutrophil-driven inflammation have emerged as consistent hallmarks of severe COVID-19 (3), while persistent myeloid activation and “trained immunity” signatures are increasingly linked to postacute sequelae (4).

Signal regulatory protein α (SIRPα) is a biologically and clinically relevant imaging target in this context. A membrane-bound immunoglobulin superfamily receptor, SIRPα is constitutively expressed across myeloid populations—monocytes, macrophages, dendritic cells, and neutrophils (5)—with inducible expression on natural killer cells under activation conditions (6). Its best-characterized function is the interaction with CD47 protein, a broadly expressed “self-marker” that delivers the inhibitory “don’t-eat-me” signal to prevent phagocytosis of healthy cells. This immune checkpoint helps balance homeostasis, preventing excessive clearance of normal tissue, but it also creates a barrier to antitumor immunity. Indeed, blockade of the CD47–SIRPα axis is being pursued in oncology to unleash macrophage-mediated clearance of tumor cells (5). What distinguishes SIRPα from TSPO or [^18^F]FDG is its lineage specificity and regulatory positioning. Rather than reflecting a generic stress response, SIRPα is tightly linked to myeloid immune regulation, particularly in secondary lymphoid organs (lymph nodes, spleen, bone marrow), where systemic orchestration of immunity occurs (5). Importantly, in human lung tissue, SIRPα expression is confined to alveolar macrophages and absent from epithelial and endothelial compartments (7). This cell-type restriction provides a clearer interpretive link to myeloid biology compared with [^18^F]FDG, which reflects nonspecific metabolic activity, or TSPO, which is expressed across multiple innate and stromal lineages.

Within this context, Stammes et al. (8) reported the application of SIRPα-directed immuno-PET in severe acute respiratory syndrome coronavirus 2 (SARS-CoV-2) viral infection. The authors reported the use of a nanobody-based PET tracer directed against SIRPα—[^64^Cu]Cu-SIRPα-Nb—to image myeloid cells during experimental SARS-CoV-2 infection in cynomolgus macaques. To demonstrate the specificity, the authors performed binding assays in both human and nonhuman primate peripheral blood mononuclear cells, quantifying selective interaction with CD14^+^ monocytes, as well as in engineered SIRPα-expressing versus wild-type HT1080 cells. In vivo tracer uptake was tracked longitudinally, with PET scans at multiple time points up to 52 d after infection. The authors also profiled immune composition in blood samples, revealing shifts in lymphocyte-to-monocyte ratios, but without macrophage-specific phenotyping. Together, these approaches provided molecular and cellular support for tracer specificity, although questions remain about whether the relatively low PET signal magnitude will allow detection of more nuanced differences across the broad range of SIRPα expression.

The imaging results showed several features of myeloid biology in SARS-CoV-2 infection. [^64^Cu]Cu-SIRPα-Nb uptake increased in mediastinal lymph nodes as early as 3 d after infection, suggesting recruitment or activation of myeloid cells in draining lymphatics. By contrast, uptake in CT-identified pulmonary lesions was minimal with the SIRPα tracer but clearly detectable with the TSPO tracer [^18^F]DPA-714 (*N,N*-diethyl-2-(2-[4-(2-fluoroethoxy)phenyl]-5,7-dimethylpyrazolo[1,5-a]pyrimidin-3-yl)acetamide). This divergence highlights that these modalities are not interchangeable, as SIRPα reflects pan-myeloid distribution and TSPO integrates broader innate and stromal activation. The absence of SIRPα signal in the lungs could be a true biologic finding, reflecting compartmentalized macrophage biology, and needs further cross-validation. In noninfected nonhuman primates, baseline PET/CT scans showed low [^64^Cu]Cu-SIRPα-Nb uptake, with only physiologic signal in salivary glands and minimal tracer-positive volume in mediastinal lymph nodes, providing a quantitative and visual baseline for comparison with postinfection increases. Most strikingly, the authors observed persistent and increased uptake of the SIRPα tracer in bone marrow and spleen at later time points, extending out to 52 d. This finding suggests prolonged systemic myeloid activation. Notably, recent human studies demonstrated that SARS-CoV-2 infection can imprint long-term epigenetic programs in hematopoietic stem and progenitor cells and their myeloid progeny, leading to enhanced responsiveness months after infection (9). This phenomenon, termed “trained immunity,” has also been described in monocytes of patients with COVID-19 and proposed as a contributor to postacute COVID-19 syndrome (i.e., long COVID) (4). Although the present PET and profiled immune composition data cannot establish such links directly, the observed marrow and splenic uptake patterns are consistent with the possibility of persistent innate imprinting and warrants orthogonal validation. Furthermore, given basal SIRPα expression in many tissues and the limited sensitivity of PET relative to proteomic assays, future work should define detection thresholds and specificity across inflammatory contexts.

These results, although promising, also emphasize the interpretive boundaries of immuno-PET. Tracer uptake alone cannot distinguish protective from pathogenic myeloid activity. Macrophages and monocytes may simultaneously clear virus, promote tissue repair, and drive immunopathology. More detailed tissue and cell-level characterization is therefore required to interpret whether increased SIRPα signal reflects beneficial containment or harmful persistence. Nonhuman primates provide invaluable translational insight, given their immunologic proximity to humans, but the constraints are high: cohort sizes remain small, repeated anesthesia is stressful, terminal histologic validation is often not feasible, and ethical barriers are magnified in high-consequence infections. In this study, the disease phenotype in cynomolgus macaques was mild—consistent with prior reports for this species (10)—limiting extrapolation to severe, intensive care unit–level COVID-19, yet highly relevant for the majority of human cases, which are mild to moderate but may progress to long-term sequelae.

For nuclear medicine and immunology alike, the significance of this work lies in expanding the immuno-PET tool kit. Each tracer brings its own strengths and weaknesses. [^18^F]FDG is sensitive but nonspecific, lighting up nearly any metabolically active process. TSPO imaging provides information about innate activation but is complicated by genetic polymorphisms in binding affinity. CD8-targeted tracers provide readouts of adaptive immune activity but offer limited insight into the innate immune compartment (11). SIRPα imaging adds another layer: a pan-myeloid view that may be less specific for function but more comprehensive for distribution. Used in combination, these tracers could ultimately provide a systems-level map of immune activity, from innate to adaptive, across the whole body. The translational opportunities are considerable. Beyond infection, myeloid cells are central players in oncology, fibrosis, and autoimmunity. The SIRPα–CD47 axis is already being targeted clinically to enhance macrophage-mediated tumor clearance (12). A noninvasive imaging tool for SIRPα could help stratify patients for these therapies, monitor pharmacodynamic effects, and identify sites of off-target inflammation. In chronic lung disease or systemic autoimmunity, SIRPα imaging could quantify the burden of myeloid activation across multiple organs, guiding therapeutic interventions.

## DISCLOSURE

The laboratory of Sridhar Nimmagadda is supported by the following grants from the National Institutes of Health: R01 CA236616, R01 CA269235, and P41EB024495. No other potential conflict of interest relevant to this article was reported.
